# Prevalence of Sedentary Behavior in Older Adults: A Systematic Review

**DOI:** 10.3390/ijerph10126645

**Published:** 2013-12-02

**Authors:** Juliet A. Harvey, Sebastien F. M. Chastin, Dawn A. Skelton

**Affiliations:** School of Health Sciences, Institute of Applied Health Research, Glasgow Caledonian University, Cowcaddens Road, Glasgow G4 0BA, UK; E-Mails: Sebastien.Chastin@gcu.ac.uk (S.F.M.C.); Dawn.Skelton@gcu.ac.uk (D.A.S.)

**Keywords:** sedentary, prevalence, aging, older adults, sitting, television, review

## Abstract

Sedentary behavior is a cluster of behaviors adopted in a sitting or lying posture where little energy is being expended. Sedentary behavior is a risk factor for health independent to inactivity. Currently, there are no published systematic reviews on the prevalence of sedentary behavior objectively measured in, or subjectively reported by, older adults. The aim of this systematic review was to collect and analyze published literature relating to reported prevalence of sedentary behavior, written in English, on human adults, where subjects aged 60 years and over were represented in the study. 23 reports covered data from 18 surveys sourced from seven countries. It was noted that sedentary behavior is defined in different ways by each survey. The majority of surveys included used self-report as a measurement of sedentary behavior. Objective measurements were also captured with the use of body worn accelerometers. Whether measurements are subjective or objective, the majority of older adults are sedentary. Almost 60% of older adult’s reported sitting for more than 4 h per day, 65% sit in front of a screen for more than 3 h daily and over 55% report watching more than 2 h of TV. However, when measured objectively in a small survey, it was found that 67% of the older population were sedentary for more than 8.5 h daily.

Received: 12 September 2013; in revised form: 1 November 2013 / Accepted: 18 November 2013 / Published:

## 1. Introduction

It is well known that physical activity (PA) is an influencing factor for healthy aging and lack of PA has been associated with chronic disease [1]. Recently, an emergence of research in sedentary behavior (SB) has indicated that SB is an independent health risk factor, separate to lack of PA, associated with successful aging, morbidity and mortality [2,3,4]. The amount of time spent being sedentary is an important risk factor associated with several aspects of ill health, including overweight and obesity and associated metabolic diseases [5]. Therefore, both SB and PA are important factors to consider in the health of older adults. Further work is required in this area with regard to cause of these outcomes and potential confounding factors. At this stage it is important to define the current level of SB to allow for the size of the issue to be identified.

Generally, the older adult section of society is underserved in physical activity and sedentary behavior research [6]. Although there is a fair amount of research on the sedentary behavior of children and young adults, there is little on older people to allow policy recommendations to give detailed information on reducing sedentary behavior in older adults [5,7,8,9,10,11].

The definition of SB has been under scrutiny lately, but general consensus indicates that it should be defined by both posture and low energy expenditure (<1.5 METS) during waking hours and includes activities such as watching television, computer use and travel [12]. The aim of this systematic review was to synthesize evidence from large scale studies, relating to the prevalence of sedentary behavior, published in English, on human adults, where subjects aged 60 years and over were represented in the study.

## 2. Experimental Section

### 2.1. Search Strategy

With the assistance of a medical librarian, a literature search was conducted using electronic databases in August 2013: The Allied and Complementary Medicine Database (AMED); Cumulative Index of Nursing and Allied Health Literature (CINAHL); Medline. Using a Boolean search strategy, keywords were entered relating to the definition of sedentary behavior e.g., sedentary**;** inactivity; activity restriction; sitting; television; screen time and computer use and pertinent search terms from each sites thesaurus (for full search details see Table S1, Table S2 and Table S3 in Supplementary File). A manual search of key journals within the area of aging and physical activity was also conducted along with checking reference lists of the selected papers. Authors of included papers were contacted requesting any additional data sources.

### 2.2. Inclusion and Exclusion Criteria

Included papers were published in English, in scholarly journals, included adult human subjects, with no restriction on publication date. The papers were reviewed by three independent researchers using inclusion and exclusion criteria (Table 1). The papers were reviewed systematically, excluding irrelevant papers, starting with consideration of the paper’s title. At the next stage, abstracts were considered and then the full texts of the remaining papers were reviewed. The papers that were not excluded were included in the review. Where conflicting opinions arose with regard to the inclusion decision the paper was included to the next phase for further consideration and excluded when there was agreement on the inclusion of said paper.

**Table 1:** Inclusion and exclusion criteria for systematic review.

Inclusion	Exclusion
Participants over 60 years of age. The study had to be designed to examine the prevalence of sedentary behavior in a population of over 200 subjects, where sedentary behavior is clearly defined as an outcome measurement, *i.e.*, not merely a lack of physical activity. The recording period should be for at least one day.	In-lab setting. Insufficient detail on older adults. Method studies. Reports published in books, conferences, posters, thesis. Measurement via mechanical pedometers. Wheelchair activity. Abstract where full texts were not available.

### 2.3. Methods of Data Presentation

#### 2.3.1. The Older Adult

There is not a consistent definition of older adults in the included papers. Over 60 years and over 65 years are the most frequently occurring definitions. Some papers included older adults as a group within a larger scale population study; some were specifically designed to describe the behavior of the older population. To be inclusive, papers were included where data from participants over 60 years were included in the study.

#### 2.3.2. Sedentary Behavior

The definition of sedentary behavior was not standardized until 2012 [12], therefore there is wide range of assessment and analysis of sedentary behavior in the reviewed literature. Measurement is by posture, energy expenditure or activity domain. Some papers represent more than one report of sedentary behavior. When examining the amount of sedentary behavior in older adults most surveys report by subjective self-report, e.g., asking about TV viewing, screen use, sitting time; or objectively by accelerometer. In this age group there was little difference in results whether the participants’ were asked to report weekday sitting and weekend sitting.

#### 2.3.3. Compiling, Weighting and Pooling the Data

Different measurement methods and categorizations were used to define sedentary behavior in the included studies. Measurement of sedentary behavior is defined by time (minutes or hours) in a week, or day. Hours per day was selected as the common scale to assimilate and allow comparison of data across the studies. Therefore, where the account of sedentary behavior is reported by week, the number is divided by 7 to give an average daily measurement. This method had been used in several papers and shown to have good reliability as a criterion measure [13]. Where papers provided separate reports by gender, this has been included, but in addition, an average of both has been calculated to allow a wider comparison across studies.

To allow summations between studies the population data has been presented as a percentage of the total population of older adults. Prevalence data were pooled into meta-prevalence using the inverse variance method. For total sitting time, the data were pooled from 7/9 surveys. The data from Katzmarzyk *et al.* [14] was excluded because of the scale used, along with the data from Patel *et al.* [15], due to the SB measurement description. The results of these papers are reported separately in the results section. For TV viewing time data were pooled from 9/10 surveys. The data from Wijndaele *et al.* [16] was excluded due to reporting scale used and is presented separately. Screen time data was pooled from 3 studies for meta-prevalence. There was not sufficient data to carry out inverse variance on computer use or sedentary time by accelerometry, so they are presented and described as per the papers.

## 3. Results

### 3.1. Selection Process Results

**Figure 1:**
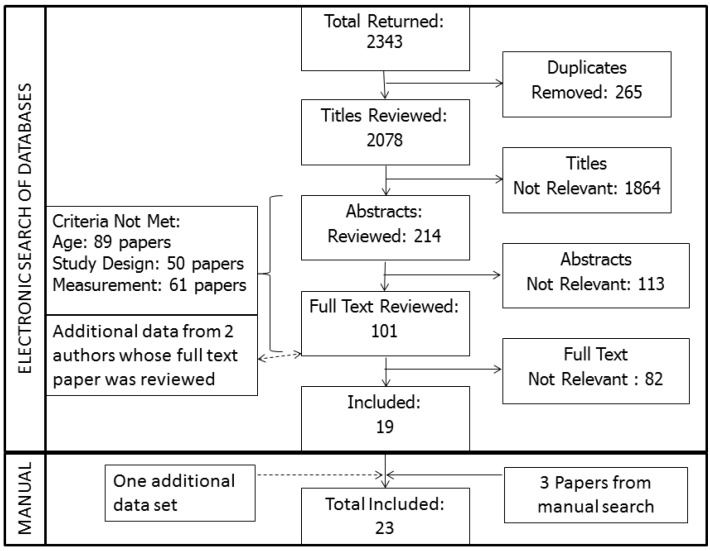
Prisma diagram of the systematic process of identification of relevant literature.

The electronic search returned a total of 2,343 papers (Medline: 732; AMED: 722; CINAHL: 888). After removal of duplicated papers, 2,078 papers were reviewed. The title review excluded 1,864 papers, the abstract review excluded 113 and the full text excluded 82, leaving 19 papers to be included for the study. At the stage of reviewing the full texts, where it was felt that additional information was needed about a particular study, the author was contacted once by e-mail requesting further information. Two included authors (Dunstan [17] and Banks [18]) provided further data sets to support their paper. One more data set was sourced and three papers were highlighted by manual searching. The process of the review resulted in 23 reports of prevalence of sedentary behavior in older adults as illustrated in Figure 1.

### 3.2. Information Sources

**Figure 2:**
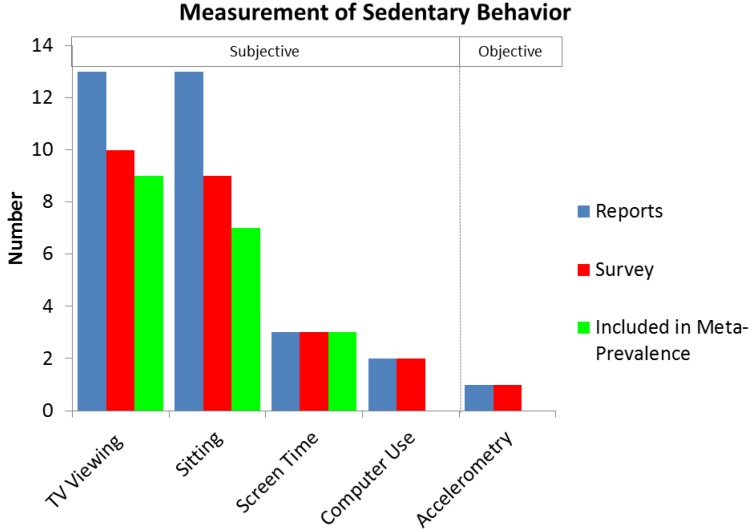
Range of sedentary behavior measurements employed when reporting prevalence of sedentary behavior in older adults, along with generation of results. Note that some surveys report more than one domain of sedentary behavior.

As can be seen from Figure 2, there were 13 papers on sitting, covering nine surveys with seven studies used for the meta-prevalence. With TV viewing 13 papers, covering 10 surveys, nine of which were included in the meta-prevalence. Three papers covered three studies, all of which were included in the meta-prevalence for screen time. Two surveys covered two surveys on computer use and one paper covered 1 survey on accelerometry.

### 3.3. Sedentary Behavior Surveys

The identified papers came mostly from developed countries: Australia (4); Canada (3); Japan (2); Norway (1); Spain (2); UK (4); USA (7), and covered information from 18 surveys and reported sedentary behavior of over 500,000 older adults. Table 2 shows the country of origin and survey details of each of the included papers.

**Table 2:** Reporting of prevalence of sedentary behavior in community dwelling older adults: Survey detail and paper detail.

Country	Survey Name (Study Period) *Study Question: Recollection Period*; *Reporting Period*; *Domain.*	Authors (published date) N Older Adult (OA)/ *total N*, *if appropriate* OA Mean Age, Men (%) Measurement
**Australia**	* The Australian Diabetes Obesity and Lifestyle study (Aus Diab) (2004–2005) *Face to Face Interview: Typical; Week; TV and Sitting.*	Dunstan *et al.* (2010) [17] (Supplementary Data Provided) 1,703 > 65 years Aged: 73.1 years; ♂ 47.5% Data: Weekly TV and sitting in quartiles with gender.
	* New South Wales Physical Activity Survey (NSW PAS) (1996) *Telephone Interview: Typical; Week; TV.*	Salmon *et al.* (2000) [19] 882 > 60 years/3,392 >18 years Mean age unknown for OA; ♂ 46.8% Data: Daily TV in 4 time categories.
	* 45 and Up Study (2006–2008) *Postal Questionnaire: Typical; Daily; Sitting and TV watching or using a computer.*	Banks *et al.* (2011) [18] (Supplementary Data Provided) 103,255 > 65 years Aged: 74.6 years; ♂ 51.7% Data: Daily screen time and sitting in 5 time categories with gender.
	* 45 and Up Study (2006–2008) *Postal Questionnaire: Typical; Daily; Sitting.*	Van der Ploeg *et al.* (2012) [20] 80,547 > 65 years/222,497 > 45 years Age Groups: 65–74 years, >75 years; ♂ 47.6% Data: Daily sitting in 4 time categories with 2 age groups
**Canada**	* Canadian Community Health Survey (CCHS) (2008–2009) *Survey Collection Method Unknown; Daily; Sitting.*	Dogra and Stathokostas (2012) [3] 9,478 > 65 years/19,538 > 45 years Mean age not known for OA; ♂ 44.8% Data: Daily sitting in 3 time categories with gender.
	* Canadian Community Health Survey (CCHS) (2007) *Structured Interview: 3 months; Typical Week; TV and Computer.*	Shields and Tremblay (2008) [21] 6,742 >65 years/57,617>20 years Mean age and % men not known for OA Data: Frequent TV and computer use presented as one time category with gender and age group.
	* Canadian Fitness Survey (CFS) (1981, baseline) *Structured Interview: Most Days; Daily; Sitting.*	Katzmarzyk *et al.* (2009) [14] 3,375 > 60 years/17,013 > 18 years Age and % men not known Data: Daily sitting in 5 time categories.
**Japan**	* Unknown Name (February to March, 2010) *Postal Questionnaire: Average; Daily and Frequency of Viewing in a Week; TV.*	Ionue *et al.* (2012) [22] 1,806 > 65 years (up to 74) Aged: 69.2 years; ♂ 51.1% Data: Weekly TV by mean, 25th and 75th percentile.
	* Unknown Name (February to March, 2010) *Postal Questionnaire: Average; Daily and Frequency of Viewing in a Week; TV.*	Kikuchi *et al.* (2013) [23] 1,665 > 65 years (up to 74) Aged: 69.5 years; ♂ 52% Data: Weekly TV in 2 time categories with gender
**Norway**	** Nord-Trøndelag Health Survey 3 (HUNT 3) (2006–2008) *Self-Completing Questionnaire: Daily; Sitting, TV viewing in categories.*	Chau *et al.* (2013) [24] 13,433 Sitting and 15,173 TV > 60 years Mean age and % men not known for OA Data: Sitting in 4 time categories; TV viewing in 3 time categories.
**Spain**	** Unknown Name (Baseline 2001) *Structured Home Interview: Daily; Sitting asked for a weekday and weekend.*	Balboa-Castillo *et al.* (2011) [25] 1,097 > 62 years Aged: 70.3 years; ♂ 50.8% Data: Daily sitting by mean, 25th and 75th percentile.
	** Unknown Name (2001 and 2003) *Structured Home Interview (2001); Telephone Interview (2003): Daily; Sitting asked for a weekday and weekend.*	León-Muńoz *et al.* (2013) [26] 2,635 > 60 years Aged: 71.3 years; ♂ 43.6% Data: Sedentariness over time between 2001 and 2003 in 4 categories.
**UK (England and Scotland)**	** European Prospective Investigation into Cancer—Norfolk, England. (EPIC) (Baseline 1998) *Self-Completing Questionnaire: Question not described.*	Wijndaele *et al.* (2011) [16] 12,608 OA Aged: 61.4 years; ♂43.4% Data: Daily TV viewing in tertiles with gender.
	* Health Survey England (HSE) (2008) *Self-Completing: last 4 weeks; frequency and duration; TV sitting and non-TV sitting; Accelerometry: 7 days with at least one valid day, <100 counts = SB.*	Stamatakis *et al.* (2012) [27] 2,765 > 60 years with 649 accelerometry Aged: 73.2 years; ♂ 45.4% (44.9% in accelerometry group) Data: Daily sedentary time by self-report and accelerometry in tertiles.
	* Scottish Health Survey (SHS) (June 2003–December 2004) *Face To Face Interview: Typical day (week, then weekend); daily; TV and Other Screens.*	Scottish Government (2005) [28] 1,627 > 65 years/8,148 > 18 years Age Groups: 65–74 years, >75 years; ♂ 37.7% Data: Daily screen time, weekday/weekend day, in 5 time categories, with gender.
	** English Longitudinal Study of Ageing (ELSA) (2008–2009) *Face To Face Interview: Ordinary week (weekday summed, weekend days summed)/7; TV. Asked if they use a computer for internet or e-mails.*	Hamer and Stamatakis (2013) [29] 6,228 > 60 years Aged: 64.9 years; ♂ 45.7% Data: Daily TV time in 4 time categories; Internet use in 2 categories (yes/no)
**USA**	** American Cancer Society Cancer Prevention Study II Nutritional Cohort (ACS II NC) (Baseline 1992) *Postal Questionnaire: Past Year; Daily; Sitting.*	Patel *et al.* (2010) [15] 123,216 > 50 years Aged: 63.1 years; ♂ 43.4 Data: Daily sitting, 3 time categories, with gender.
	* US Department of Agriculture Continuing Survey of Food Intake by Individuals (DACSFII) (1994–1996) *Face To Face Interview: 0-24 h; Daily; TV. (asked on 2 occasions)*	Bowman, (2006) [30] 1,428 > 66 years / 9,157 > 20 years Mean age and % male of OA unknown Data: Daily TV, 1 time category.
	* National for Institute American Association of Retired Persons Health Diet and Health Study (NIH–AARP) (Baseline: 1995–1996 population characteristics/1996–1997 sitting) *Postal Questionnaire: Past Year; Daily; TV and Sitting. In addition, George et al. (2010)* [29] *reports—Activity in a routine day, with one sedentary response of “sitting all day”.*	George *et al.* (2010) [31] 97,039 > 50 years Aged: 63 years (age adj); ♂ 0 Data: Daily TV and sitting in 2 time categories each. Separately, “sitting all day” number.
		George *et al.* (2011) [32] 1,206 > 50 years Aged: 63 years; ♂ 75.0% Data: Daily TV and sitting in 5 time categories each, with gender.
		Matthews *et al.* (2012) [33] 240,819 > 60 years Aged: 62.3 years; ♂ 54.9% Data: Daily TV and sitting in 5 time categories each with gender.
	* National Health and Nutrition Examination Survey (NHANES) (2003–2004, 2005–2006) *Interview: Last 30 day; Daily; TV.*	Clark *et al.* (2011) [34] 2,303 > 60 years/5,738 Age: 70.9 years; % male unknown for OA Data: Daily TV given in 1 time category.
	* National Health and Nutrition Examination Survey (NHANES) (1999–2002) *Interview: Last 30 day; Daily; TV and computer use outside work.*	Ford (2012) [35] 542 deceased at follow up in 2006 Age: 66.9 years; ♂ 64.3% Data: Screen time given in 6 time categories.

Notes: * Cross-Sectional Analysis of Sedentary Time; ** Baseline of Prospective Study.

### 3.4. Findings on Prevalence of Sedentary Behavior in Older Adults

#### 3.4.1. Prevalence of Sedentary Behavior in Older Adults, by Self Report of Sitting

**Figure 3:**
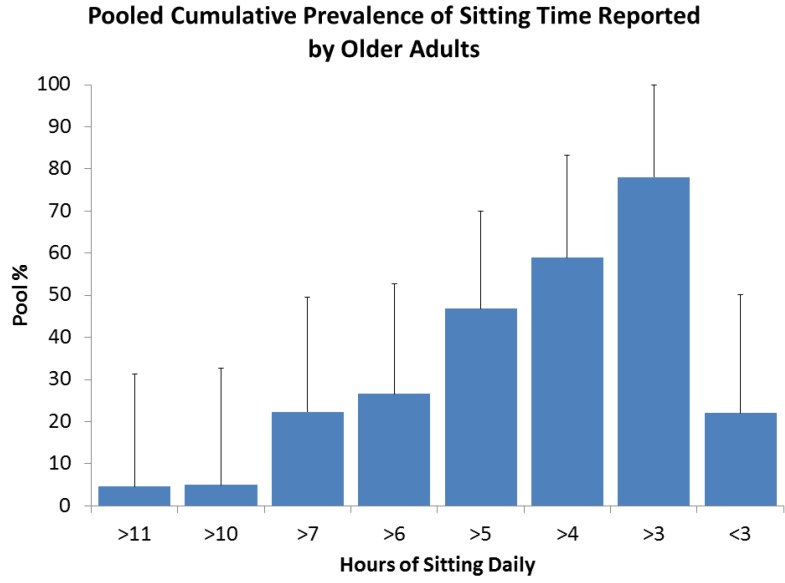
Meta-Prevalence of sitting, *N* = 372,550 older adults, *n* = 7 from six countries, note time categories of <3 h (22%) and >3 h (78%) is equal to 100%.

Sitting time data have been combined and weighted to provide meta-prevalence of time spent sitting daily by older adults, which is presented in Figure 3. These results were obtained for a total population of 372,550 compiled from seven surveys covering six countries (Australia, Canada, Norway, Spain, UK and USA) [3,17,18,24,25,27,33].

On average across the studies, 58.9% of older adults reported sitting for over 4 h, 26.6% reported over 6 h and 5.0% reported over 10 h per day. Patel *et al.* [15] using ACS II NC data, reported “*leisure time sitting*” and reported a level of 54.1% reporting over 3 h at this level, compared to 78.0% “*total sitting time*” found by the meta-prevalence method. The CFS was described by Katzmarksky *et al.* [14] employing a different question format asking participants about their sitting behavior in fractions of a day. They reported that 30.7% of older adults reported spending a half day sitting and 6.4% reported sitting all day. This is comparable to 7.9% of females reported “sitting all day” reported by George *et al.* [31]. León-Muńoz *et al.* [26] tracked changes in sedentary time over 2 years in those over 60 years of age and found that over this period 22.9% remained sedentary, 21.5% became more sedentary and 20.2% reduced their sedentary time.

There is less data available on gender and age in relation to sedentary behavior by report of sitting. It would appear there is little difference in report by gender (1.6 ± 1.6%) [3,17,18]. On average 61.5% females reported more than 4 h of sitting compared to males at 63.5% [3,17,18]. Van der Ploeg [20] reported the prevalence of sitting by age group and report 72.7% in the 65–74 years sit over 4 h per day and 76.2% in the over 75 group.

#### 3.4.2. Prevalence of Sedentary Behavior in Older Adults, by Self Report of TV Viewing

**Figure 4:**
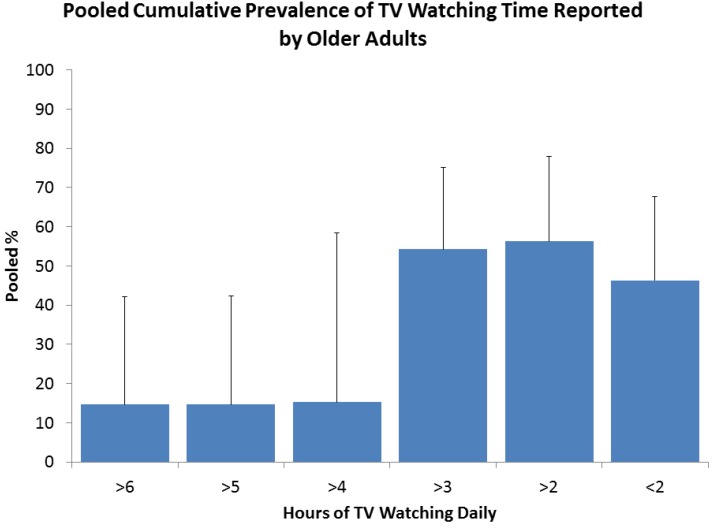
Prevalence of sitting watching TV, *N* = 275,344 older adults, *n* = 9, note the sum of <2 h (44%) and >2 h (56%) is equal to l00%.

TV viewing serves as a proxy for sedentary behavior, Figure 4 shows the meta-prevalence of TV viewing reported by older adults. These results are reported for a total population of 275,344 older adults, representing nine surveys combining data from Australia, Canada, Japan, Norway, USA and UK [17,19,21,23,24,29,30,33,34]. Fifty six percent of the older population report watching over 2 h of TV per day, 54.2% report over 3 h and 15.1% report over 4 h. The EPIC survey reported by tertiles and found that 32.5% reported TV viewing over 3.6 h per day [16].

Report of TV viewing by gender was less common, the average difference between gender report was 1.8 ± 1.4% [16,17,21,23]. The largest gender difference reported by Wijndaele, *et al.* [16] where 3.7% more females reported levels of >3.6 h TV viewing daily. Only Shields and Tremblay [21] reported TV viewing by age groups, in this instance 52.1% of individuals over the age of 75 years report over 2 h of TV watching compared to 46.9% in the 65–74 year group.

#### 3.4.3. Prevalence of Sedentary Behavior in Older Adults, by Computer Use and Screen Time

**Figure 5:**
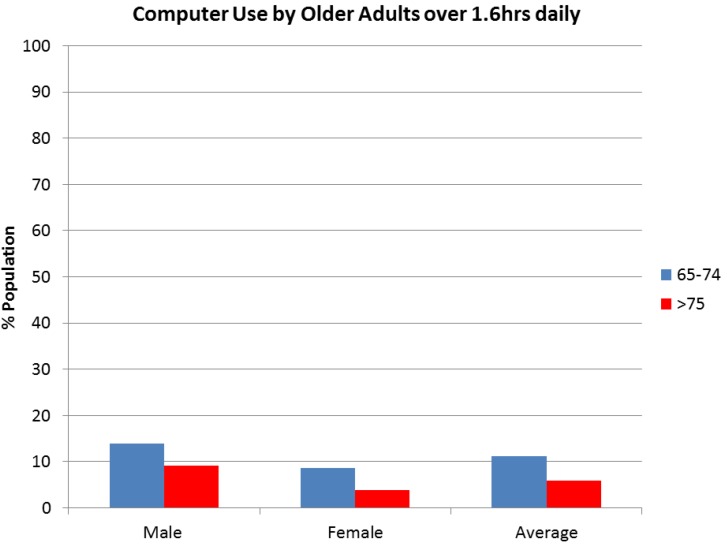
Prevalence of computer use over 1.6 h daily reported by 6,742 older adults.

Hamer and Stamatakis [29] reported that 64.6% of older adults in the UK use a computer. Shields and Tremblay [21] provided data on computer use by Canadian older adults, including factors of age and gender for 6,742 participants (Figure 5). Computer use in Canada is relatively low in older adults.Less than 10% of the average older adult uses a computer for more than 1.6hrs daily. Younger older adults use computers more than those over 74 years and the activity is more favored by males.

**Figure 6:**
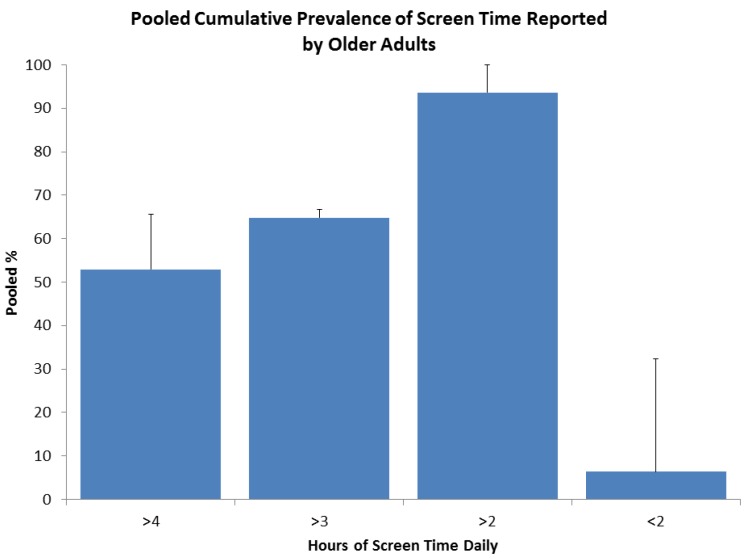
Meta-prevalence of screen time older adults, *N* = 105,424, *n* = 3, note <2 (6%) and >2 (94%) = 100%.

When computer use and TV time is combined it is termed screen time. As can be seen from Figure 6, 52.9% of the population in Australia, UK and USA reported more than 4 h daily and 93.6% reported screen time of over 2 h [18,28,35].

Bank *et al.* [18] and the Scottish Government [28] report gender specific data, there is little effect of gender with the mean difference between males and females in these two data sets (1.5 ± 1.2%). As with computer use patterns, and likely because of computer use, screen time is reported to be slightly higher in the 65–74 age group, than the over 75 years, 52.5% and 47.8%, respectively report over 4 h of screen time [28].

#### 3.4.4. Prevalence of Sedentary Behavior by Accelerometry

**Figure 7:**
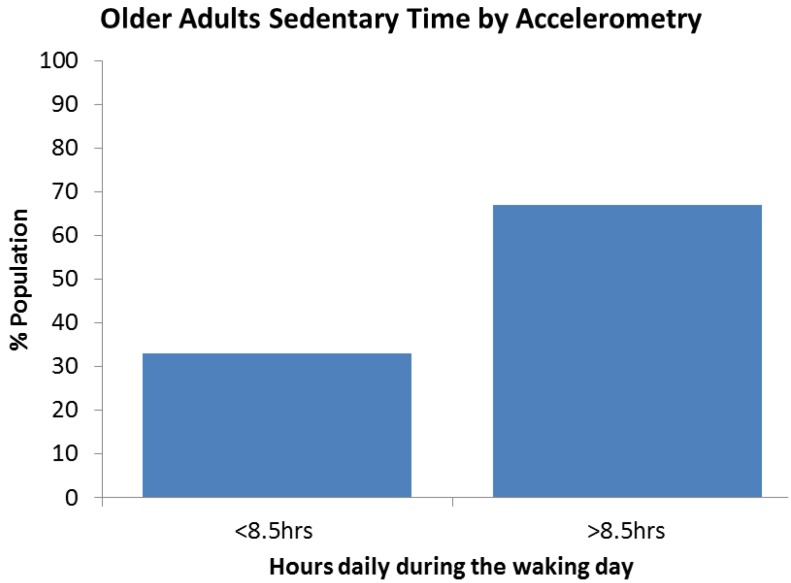
Prevalence of sedentary behavior by accelerometry in 649 older adults.

Stamatakis *et al.* [27] was the only paper to define the prevalence of objective measurement of sedentary behavior in older adults, by accelerometry using activity count. Sixty seven percent of older adults spend over 8.5 h of their waking day sitting or in low energy expenditure. Figure 7 represents the results of 649 English older adults.

## 4. Discussion

### 4.1. Summary of Findings

Globally, almost 60% of older adult’s report sitting for more than 4 h per day. When objectively measured it is found that 67% of the population are sedentary for more than 8.5 h in their waking day. Both screen time and TV time can be used as proxy measurements of sedentary behavior. When screen time is reported, 53% of older adults report sitting in front of a screen for over 4 h daily. 15% report watching more than 4 h of TV daily and 54% reported levels of 3h daily. Computers are reportedly used by 65% of older adults with fewer than 10% of older adults using a computer for over 1.6 h daily. Computer use is likely to increase with time as people become more familiar with computer technology. There is little difference in sedentary behavior trends between genders and there is a slight increase in sedentary time in the age groups over 75, compared with 65–74, with the exception of computer use, although it is worth noting there is limited specific data on the oldest old across all populations.

### 4.2. Relevance of Sedentary Behavior in Older Adults

Those individuals who are less sedentary tend to age more successfully and report better quality of life [3,25]. There has been shown to be an association between sitting time and negative health outcomes, such as increased risk of cardiovascular disease and all causes of mortality [14,24,27,33]. However, association between sitting time and invasive breast cancer risk and risk of renal cell carcinoma has been specifically examined and significant independent association was not shown, although a weak correlation could not be ruled out [31,32]. TV viewing has also been well correlated with negative health outcomes, such as cardiovascular disease, mortality and being overweight or obese [16,19,22,30]. TV viewing is also associated with other unhealthy habits such as consumption of unhealthy food and drinks or the influence of adverting to encourage these behaviors, therefore may also be a confounding factor with negative health effects of sitting [36].

### 4.3. Measurement of Sedentary Behavior in Surveys

Sedentary behavior has been measured in older adults in a number of ways to define its prevalence in a population. There is a large difference between subjective and objective reporting of sedentary behavior. When objectively measured by accelerometry 2/3 from one small study showed older populations are sedentary for more than 8.5 h daily [27]. The accelerometer employed in this case measures lack of counts of activity as sedentary, rather than sitting, thus there could potentially be some standing time included in this sedentary data. However, this is still a large difference compared to self-report, but, considering the size of the sample in this case caution should be shown when generalizing to the general population.

Self-report measures are known to have poor accuracy and lead to an underestimation of the strength of the relationship [37]. It is noteworthy that Stamatakis *et al.* [27] also reports a better association with self-report sedentary behavior and cardio-metabolic risk markers of diabetes, over objective measurement (1.074, *p* < 0.01, compared to 1.095, *p* = 0.07). In addition, Matthews *et al.* [33] notes a stronger association with mortality and TV viewing than of reports of “overall sitting”. This is likely to be related to the validity of the recording instrument, as asking about overall sitting requires more cognitive processing than asking about TV viewing. TV viewing is more easily calculable by summing TV program durations. Standardization and specification of the measurement of sedentary behavior requires more work. The accuracy of older adults self-reporting sitting time may be more accurate if researchers clarify the sitting domains, ie provide examples relevant to older adults and suggest strategies for formulating responses [38].

### 4.4. Limitations

The meta-prevalence computations were based on the inverse variance method. This method has some limitations for very high and very low prevalence. Studies with low and high prevalence in a category tend to be given an overestimated weight [39]. In addition, the studies pooled had different categories of prevalence and while we only considered non overlapping categories it is possible that weighting might have been underestimated for some studies evaluating prevalence of larger categories of time. The accelerometry data for older adults was based on a study of 649 and a single country, therefore perhaps not generalizable to the population.

### 4.5. Future Work

With many current health and physical activity policy recommendations suggesting that all older adults should minimize the amount of time spent being sedentary (sitting) for extended periods [5,7,8,9,10,11], it is vital that more is learnt about the measurement and effect of sedentary behavior.

## 5. Conclusions

Whether measurements are subjective or objective, the majority of older adults are sedentary. Approximately 60% of older adult’s report sitting for more than 4 h per day and over 54% report watching more than 3 h of TV and 65% sit in front of a screen for over 3 h. When objectively measured, only 33% of the population are sedentary for less than 8.5 h in their waking day. However, fewer than 10% of older adults report using a computer for over 1.6 h daily. There is little difference between genders and there is a slight increase in the prevalence of sedentary activity with age, with the exception of computer use. These findings suggest that sedentary behavior is very prevalent in older adults.

## Supplementary Files

Supplementary File 1 Supplementary Information (PDF, 86 KB)
