# Supplementary material for: Prevalence of Sedentary Behavior in Older Adults: A Systematic Review
Source: Int J Environ Res Public Health. 2013 Dec 2;10(12):6645–61. doi: 10.3390/ijerph10126645 (PMC3881132; doi:10.3390/ijerph10126645)
Supplement: Supplementary File 1 — Supplementary Information (PDF, 86 KB) [file ijerph-10-06645-s001.pdf]

Supplementary Information

Search Strategy

Table S1. AMED search terms, 9:03:07 a.m., Thursday, 15 August, 2013.

#	Query	Limiters/Expanders	Last Run Via	Results
S13	S9 OR S12	Search modes: Find all my search terms	Interface: EBSCOhost Research Databases<br>Search Screen: Advanced Search<br>Database: AMED—The Allied and Complementary Medicine Database	772
S12	S10 AND S11	Search modes: Find all my search terms	Interface: EBSCOhost Research Databases<br>Search Screen: Advanced Search<br>Database: AMED—The Allied and Complementary Medicine Database	38
S11	S6 OR S7 OR S8 OR S9	Search modes: Find all my search terms	Interface: EBSCOhost Research Databases<br>Search Screen: Advanced Search<br>Database: AMED—The Allied and Complementary Medicine Database	2,502
S10	S2 OR S3 OR S4	Search modes: Find all my search terms	Interface: EBSCOhost Research Databases<br>Search Screen: Advanced Search<br>Database: AMED—The Allied and Complementary Medicine Database	1,095
S9	Sedentary*****	Search modes: Find all my search terms	Interface: EBSCOhost Research Databases<br>Search Screen: Advanced Search<br>Database: AMED—The Allied and Complementary Medicine Database	749
S8	Activity Restriction	Search modes: Find all my search terms	Interface: EBSCOhost Research Databases<br>Search Screen: Advanced Search<br>Database: AMED—The Allied and Complementary Medicine Database	345
S7	Sitting	Search modes: Find all my search terms	Interface: EBSCOhost Research Databases<br>Search Screen: Advanced Search<br>Database: AMED—The Allied and Complementary Medicine Database	1,219
S6	Inactivity	Search modes: Find all my search terms	Interface: EBSCOhost Research Databases<br>Search Screen: Advanced Search<br>Database: AMED—The Allied and Complementary Medicine Database	253

Table S1. *Cont.*

#	Query	Limiters/Expanders	Last Run Via	Results
S5	TV	Search modes: Find all my search terms	Interface: EBSCOhost Research Databases<br>Search Screen: Advanced Search<br>Database: AMED—The Allied and Complementary Medicine Database	238
S4	Computer Use	Search modes: Find all my search terms	Interface: EBSCOhost Research Databases<br>Search Screen: Advanced Search<br>Database: AMED—The Allied and Complementary Medicine Database	920
S3	Video Games	Search modes: Find all my search terms	Interface: EBSCOhost Research Databases<br>Search Screen: Advanced Search<br>Database: AMED—The Allied and Complementary Medicine Database	68
S2	Television	Search modes: Find all my search terms	Interface: EBSCOhost Research Databases<br>Search Screen: Advanced Search<br>Database: AMED—The Allied and Complementary Medicine Database	128
S1	Screen Time	Search modes: Find all my search terms	Interface: EBSCOhost Research Databases<br>Search Screen: Advanced Search<br>Database: AMED—The Allied and Complementary Medicine Database	122

Table S2. CINAHL search terms, 05:54:17 a.m., Thursday, 15 August 2013.

#	Query	Limiters/Expanders	Last Run Via	Results
S1	(MH "Life Style, Sedentary")	Search modes: Find all my search terms	Interface: EBSCOhost Research Databases<br>Search Screen: Advanced Search<br>Database: CINAHL	2,000
S2	(MH "Television")	Search modes: Find all my search terms	I Interface: EBSCOhost Research Databases<br>Search Screen: Advanced Search<br>Database: CINAHL	5,246
S3	(MH "Video Games")	Search modes: Find all my search terms	Interface: EBSCOhost Research Databases<br>Search Screen: Advanced Search<br>Database: CINAHL	1,098
S4	"Computer Use"	Search modes: Find all my search terms	Interface: EBSCOhost Research Databases<br>Search Screen: Advanced Search<br>Database: CINAHL	475
S5	"Sedentary*"	Search modes: Find all my search terms	Interface: EBSCOhost Research Databases<br>Search Screen: Advanced Search<br>Database: CINAHL	4,461

Table S2. *Cont.*

#	Query	Limiters/Expanders	Last Run Via	Results
S6	(MH “Sitting”)	Search modes: Find all my search terms	Interface: EBSCOhost Research Databases Search Screen: Advanced Search Database: CINAHL	1,104
S7	“Inactivity”	Search modes: Find all my search terms	Interface: EBSCOhost Research Databases Search Screen: Advanced Search Database: CINAHL	1,668
S8	“activity restriction”	Search modes: Find all my search terms	Interface: EBSCOhost Research Databases Search Screen: Advanced Search Database: CINAHL	142
S9	“screen time”	Search modes: Find all my search terms	Interface: EBSCOhost Research Databases Search Screen: Advanced Search Database: CINAHL	153
S10	S1 OR S2 OR S3 OR S4	Search modes: Find all my search terms	Interface: EBSCOhost Research Databases Search Screen: Advanced Search Database: CINAHL	8,464
S11	S5 OR S6 OR S7 OR S8 OR S9	Search modes: Find all my search terms	Interface: EBSCOhost Research Databases Search Screen: Advanced Search Database: CINAHL	7,084
S12	S10 AND S11	Search modes: Find all my search terms	Interface: EBSCOhost Research Databases Search Screen: Advanced Search Database: CINAHL	2,141
S13	S1 OR S12	Limiters: English Language; Human; Age Groups: Aged: 65 + years, Aged, 80 and over, All Adult Search modes: Find all my search terms	Interface: EBSCOhost Research Databases Search Screen: Advanced Search Database: CINAHL	888

Table S3. Medline Search Terms, 7:36:45 a.m., Thursday, 15 August 2013.

#	Query	Limiters/Expanders	Last Run Via	Results
S12	S10 AND S11	Limiters: English Language; Human; Age Related: Aged: 65+ years, Aged, 80 and over, All Adult: 19+ years; Language: English Search modes: Find all my search terms	Interface: EBSCOhost Research Databases Search Screen: Advanced Search Database: MEDLINE	732
S11	S1 OR S2	Search modes: Find all my search terms	Interface: EBSCOhost Research Databases Search Screen: Advanced Search Database: MEDLINE	17,262
S10	S3 OR S4 OR S5 OR S6 OR S7 OR S8 OR S9	Search modes: Find all my search terms	Interface: EBSCOhost Research Databases Search Screen: Advanced Search Database: MEDLINE	36,608
S9	"Sitting"	Search modes: Find all my search terms	Interface: EBSCOhost Research Databases Search Screen: Advanced Search Database: MEDLINE	14,154
S8	"Inactivity"	Search modes: Find all my search terms	Interface: EBSCOhost Research Databases Search Screen: Advanced Search Database: MEDLINE	8,548
S7	"Computer Use"	Search modes: Find all my search terms	Interface: EBSCOhost Research Databases Search Screen: Advanced Search Database: MEDLINE	1,037
S6	(MH "Video Games")	Search modes: Find all my search terms	Interface: EBSCOhost Research Databases Search Screen: Advanced Search Database: MEDLINE	1,783
S5	(MH "Television")	Search modes: Find all my search terms	Interface: EBSCOhost Research Databases Search Screen: Advanced Search Database: MEDLINE	11,049
S4	"Activity Restriction"	Search modes: Find all my search terms	Interface: EBSCOhost Research Databases Search Screen: Advanced Search Database: MEDLINE	366
S3	"Screen Time"	Search modes: Find all my search terms	Interface: EBSCOhost Research Databases Search Screen: Advanced Search Database: MEDLINE	447

Table S3. *Cont.*

#	Query	Limiters/Expanders	Last Run Via	Results
S2	"Sedentary*"	Search modes: Find all my search terms	Interface: EBSCOhost Research Databases<br>Search Screen: Advanced Search<br>Database: MEDLINE	17,262
S1	(MH "Sedentary Lifestyle")	Search modes: Find all my search terms	Interface: EBSCOhost Research Databases<br>Search Screen: Advanced Search<br>Database: MEDLINE	1,919

© 2013 by the authors; licensee MDPI, Basel, Switzerland. This article is an open access article distributed under the terms and conditions of the Creative Commons Attribution license (http://creativecommons.org/licenses/by/3.0/).
